# Tetradecanuclear {Ni_9_Ln_5_} Clusters Featuring Encapsulated {Ln_5_} Trigonal Bipyramids: Modular 3*d*‐4*f* Architectures With Broad‐Range Magnetocaloric Response

**DOI:** 10.1002/chem.71250

**Published:** 2026-06-08

**Authors:** Thomais G. Tziotzi, Evangelia G. Asmargiannaki, Ben Thamm, Scott J. Dalgarno, Emmanouil K. Charkiolakis, Marc Ubach I. Cervera, David Gracia, Marco Evangelisti, Constantinos J. Milios

**Affiliations:** ^1^ Department of Chemistry The University of Crete Herakleion Greece; ^2^ Institute of Chemical Sciences Heriot‐Watt University Edinburgh UK; ^3^ Instituto De Nanociencia y Materiales De Aragón (INMA), CSIC Universidad De Zaragoza Zaragoza Spain

**Keywords:** amino acids, cluster compounds, heat capacity, heterometallic Ni^II^/Ln^III^ complexes, magnetic properties

## Abstract

The solvothermal reaction between Ni(HCOO)_2_^.^2H_2_O, 2‐aminoisobutyric acid (aibH), Ln(OAc)_3_·4H_2_O (Ln = Gd, Y, Dy) and NEt_3_ in MeOH/MeCN affords the tetradecanuclear [Ni_9_Ln_5_(OH)_9_(aib)_12_(OAc)_x_(ΗCOO)_y_]^.^12MeCN complexes (Ln = Gd, x = 6, y = 6, **1** ^.^12MeCN; Ln = Y, x = 6, y = 6, **2** ^.^12MeCN; Ln = Dy, x = 3, y = 9, **3** ^.^12MeCN) in good yields. All three clusters are isostructural, featuring a trigonal bipyramidal {Ln_5_} core encapsulated within a triply edge‐capped {[Ni^II^_3_]Ni^II^_6_} trigonal prism. Magnetic measurements reveal dominant ferromagnetic intermolecular interactions in the Gd and Dy analogues, whereas the Y derivative exhibits weak antiferromagnetic behavior. Magnetothermal and heat‐capacity measurements for **1** ^.^12MeCN indicate a significant magnetocaloric effect, reaching −∆*S*_m _= 28.1 J kg^−1^ K^−1^ for full demagnetization from *B* = 7 T at *T* = 3.9 K, while sustaining large −∆*S*_m_(*T*,∆*B*) and ∆*T*_ad_(*T*,∆*B*) responses across an unusually broad temperature range.

## Graphical abstract

The use of 2‐aminoisobutyric acid in 3d–4f chemistry affords three {Ni₉Ln_5_} clusters, with the Gd analogue exhibiting an unusually broad magnetocaloric effect across an extended temperature range.

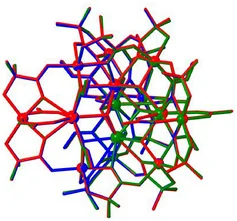

## 1 Introduction

Acquiring and maintaining very‐low temperatures has become a stringent requirement in several technical applications including MRI scanners [1, 2], electromagnetic radiation detectors [3], and quantum technologies [4, 5]. Conventional methods used to achieve such cooling power rely heavily on the use of ^3^He which is quickly becoming a scarce resource [6]. As such, recent research efforts have been directed towards the discovery of more sustainable cooling processes that are energy efficient, reliable and whose usage pose no harm to the environment.

A promising candidate is found in adiabatic demagnetisation refrigeration (ADR), which exploits the heating or cooling of magnetic materials when exposed to a varying magnetic field, the so‐called magneto‐caloric effect (MCE) [7]. At its core, the MCE refers to the change in magnetic entropy (∆*S*_m_) when a magnetic field is applied or removed (∆*B*). Under adiabatic conditions, this change in magnetic entropy is compensated for by an equal and opposite change in the temperature (∆*T*_ad_) of the magnetic material. By cyclically applying and removing the magnetic field, ADR harnesses this temperature change to achieve cooling [8]. In addition, no moving parts or non‐renewable gases are required during the refrigeration cycle, making ADR more energy efficient than conventional refrigeration technologies such as ^4^He‐^3^He dilution refrigerators [3].

To maximise MCE, the use of gadolinium has led to molecular magnetocaloric materials with great potential at very‐low temperatures [9, 10, 11]. The favourable properties of these compounds arise due to the large magnetic moment and negligible orbital angular momentum of the gadolinium atom, while the weak Gd···Gd magnetic interactions within these molecules ensure the MCE response to occur at very‐low temperatures [12]. Additionally, the use of lightweight ligands including formates, acetates and carbonates greatly reduces the amount of non‐magnetic material, which further enhances the MCE [12]. Recent reports have also shown that these materials can display a large MCE at magnetic applied fields attainable by small permanent magnets (e.g. 1–2 T) [12], displaying promising results for local refrigeration applications [13, 14].

One interesting subset of these materials are gadolinium‐containing 3d–4f molecular clusters, in which transition and gadolinium metal ions are combined to form aesthetically pleasing structures with superior magnetocaloric properties [15, 16, 17, 18, 19]. One such exciting example is the recently reported {Gd_152_Ni_14_} complex and its record‐breaking −∆*S*_m_ value of 52.65 J kg^−1^ K^−1^ at *T *= 2.5 K and ∆*B* = 7 T [18]. Similarly, a 3D Gd‐Mn MOF material has been reported to show −∆*S*_m_ of 25 J kg^−1^ K^−1^ at *T *= 2 K and ∆*B* = 1 T [15]. For both of these compounds, the large MCE is ascribed to the presence of the transition metal ions, specifically mentioning the weak Ni···Gd and Mn···Gd magnetic interactions, respectively.

These fascinating magnetocaloric properties paired with our prior success of obtaining heterometallic complexes through use of 2‐aminoisobutyric acid (aibH) [20, 21, 22, 23, 24, 25, 26, 27] have prompted us to investigate the synthesis of gadolinium‐based 3d–4f complexes with aibH. Herein, we extend the family of 3d–4f gadolinium‐based clusters and report on the synthesis of three new isostructural complexes, {Ni_9_Ln_5_} (Ln = Gd^3+^, **1**; Ln = Y^3+^, **2**; Ln = Dy^3+^, **3**), with complex **1** displaying a large magnetocaloric effect of −∆*S*_m_ = 28.1 J kg^−1 ^K^−1^ for full demagnetization from *B* = 7 T at *T* = 3.9 K.

## 2 Results and Discussion

### 2.1 Syntheses

The 1: 1: 1 solvothermal reaction of Gd(OAc)_3_·4H_2_O with Ni(HCOO)_2_·2H_2_O and aibH in MeOH/MeCN (1:1), in the presence of base, NEt_3_, forms complex [Ni_9_Gd_5_(OH)_9_(aib)_12_(OAc)_6_(ΗCOO)_6_] (**1**) in moderate‐to‐good yield, according to the following chemical Equation (1):

$$9\mathrm{Ni}{\left(\mathrm{HCOO}\right)}_{2}\cdot 2H_{2}O+5\;\mathrm{Gd}{\left(\mathrm{OAc}\right)}_{3}\cdot 4H_{2}O+12\mathrm{aibH}$$

(1) $$\begin{array}{c} \left[Ni_{9}Gd_{5}{\left(\mathrm{OH}\right)}_{9}{\left(\mathrm{aib}\right)}_{12}{\left(\mathrm{OAc}\right)}_{6}{\left(\mathrm{HCOO}\right)}_{6}\right]\\ +12\mathrm{HCO}O^{-}+9H\mathrm{OAc}+12H^{+}+29H_{2}O \end{array}$$

Given that the metals to ligand ratio found in the product is ∼2: 1: 2.5 (Ni: Gd: aib), we repeated the solvothermal reaction using the above ratio to increase the yield of the reaction, but this did not lead to a drastic change. Furthermore, we investigated various synthetic parameters, e.g. reaction time, temperature/pressure, presence/absence of base, as well as the nature of the base, finding out that: (1) complex **1** only forms under solvothermal conditions; when the reaction occurs under normal temperature and pressure conditions, we obtain an amorphous solid that we could not characterize further, (2) when we perform the reaction in the absence of base, it does not lead to the formation of any crystalline material, and (3) the presence of the formate ions is deemed essential for obtaining **1**, since employment of different nickel salts i.e., Ni(OAc)_2_^.^4H_2_O, Ni(ClO_4_)_2_^.^6H_2_O, and Ni(NO_3_)_2_^.^6H_2_O did not yield any crystalline material. Following the same synthetic route, we were able to obtain complex [Ni_9_Y_5_(OH)_9_(aib)_12_(OAc)_6_(ΗCOO)_6_] (**2**), upon replacing Gd(OAc)_3_·4H_2_O with Y(OAc)_3_·4H_2_O, while the same method upon using Dy(OAc)_3_^.^4H_2_O led to the isostructural Dy analogue, [Ni_9_Dy_5_(OH)_9_(aib)_12_(OAc)_3_(ΗCOO)_9_] (**3**), with the only difference being the presence of three acetate and nine formate ligands in **3**, instead of six acetate and six formate ligands in **1** and **2**.

### 2.2 Description of Structures

**FIGURE 1:**
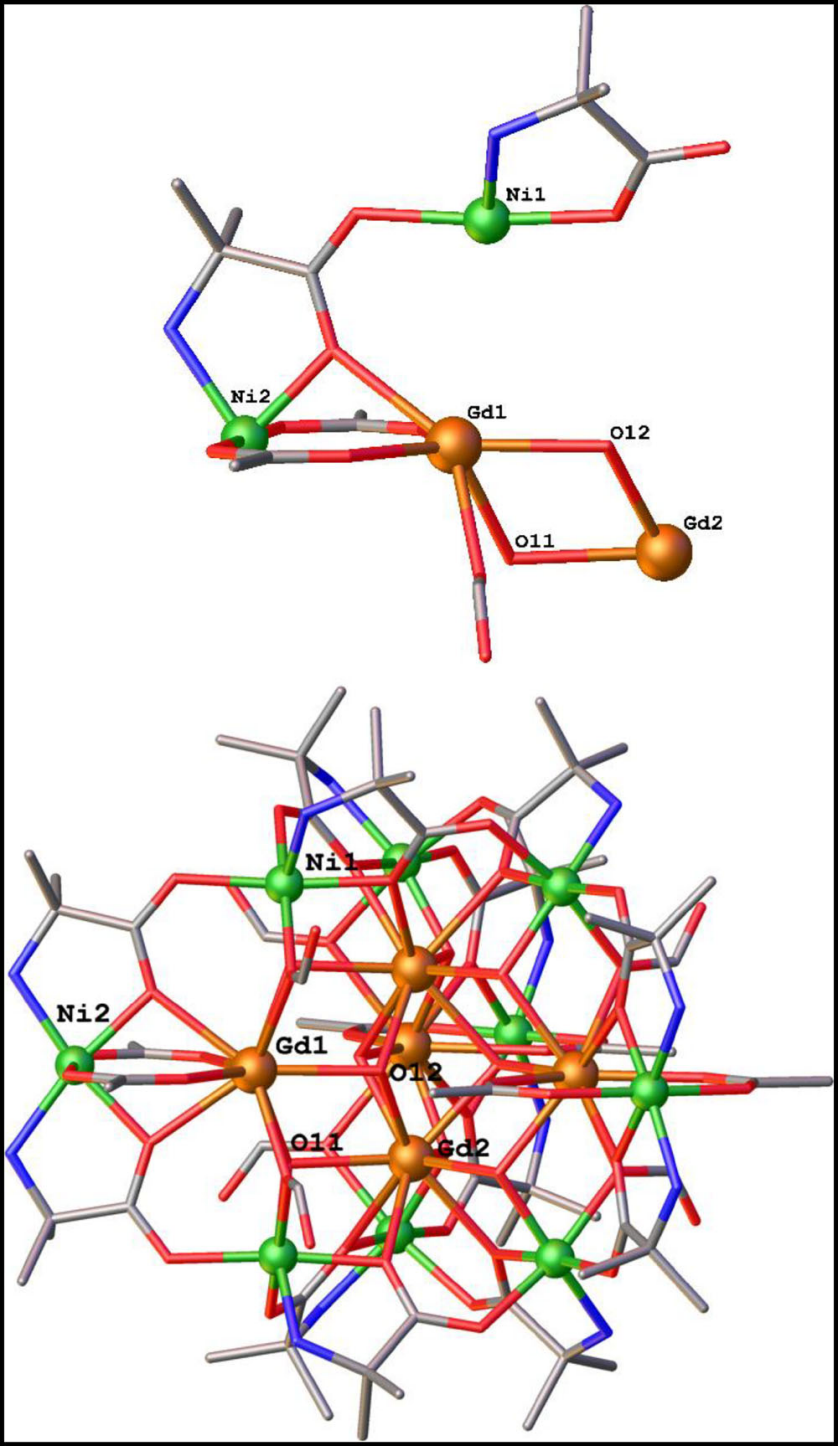
Asymmetric unit found in **1** (top) and its symmetry expanded molecular structure (bottom). Colour scheme: Gd^III^, brown; Ni^II^, green; O, red; N, blue; C, grey. Hydrogen atoms omitted for clarity.

**FIGURE 2:**
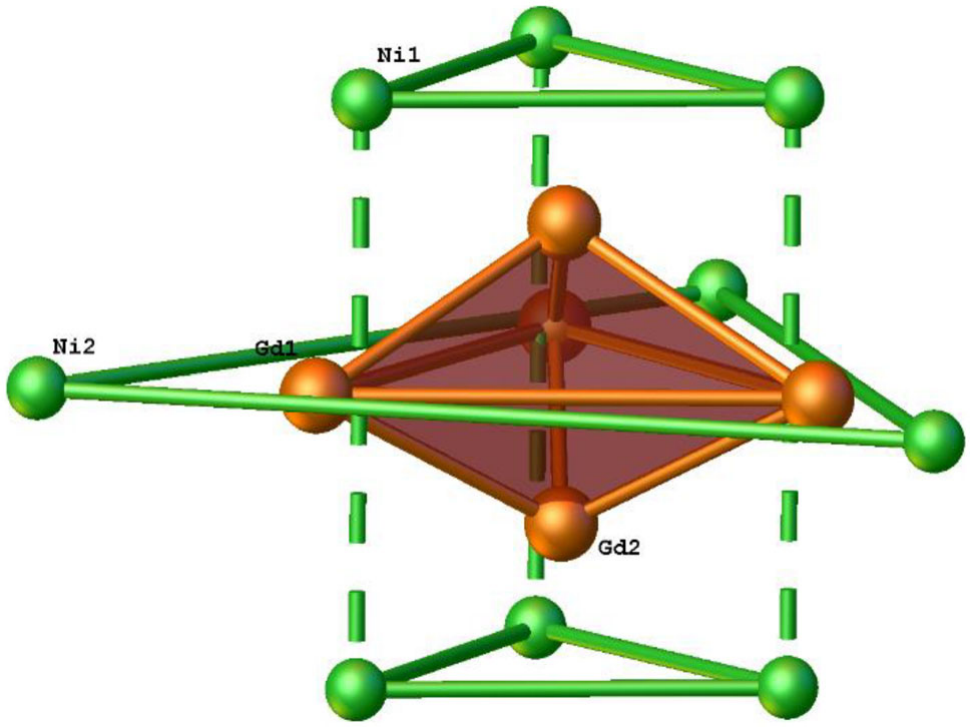
Metallic skeleton of **1** composed of a {Gd_5_} trigonal bipyramid with two {Ni_3_} triangles above and below the lanthanide centre, forming a trigonal prism, itself being triply edge‐capped. Color scheme: Gd^III^, brown; Ni^II^, green.

Complex **1** crystallizes in the hexagonal space group *P*6_3_/*m* with one‐sixth of the complex present in the asymmetric unit (Figure 1). The metallic skeleton of **1** consists of a propeller‐like {Ni_3_Gd_5_} unit located between two equilateral {Ni_3_} triangles (Ni···Ni: 5.25 Å). Alternatively, the two {Ni_3_} triangles describe a trigonal prism with the two faces separated by ∼7 Å, encapsulating a normal {Gd_5_} trigonal bipyramid, and further being triply edge‐capped, thus forming a {[Ni_6_⊂Gd_5_]Ni_3_} metallic unit (Figure 2). The Gd^…^Gd distance on the basal bipyramidal plane is ∼6 Å, while the apical centres are located 1.8 Å above and below the base, thus forming a “suppressed” trigonal bipyramid. Three μ_3_‐OH^−^ ions (O12 and s.e., BVS value of 0.84) are situated along the three edges of the basal bipyramidal plane and bridge to one basal and both apical Gd^III^ ions. The remaining six μ_3_‐OH^−^ ions (O11 and s.e., BVS value of 0.61) form two (μ_3_‐OH)_3_ planes above and below the {Gd_5_} trigonal bipyramid with each μ_3_‐OH^−^ ion bridging to one basal Gd^III^, one apical Gd^III^, and one Ni^II^ ion, thus linking the {Gd_5_} centre to the two {Ni_3_} triangles. The [Ni_6_⊂Gd_5_] encapsulation is completed by six μ_2_‐HCOO^−^ ions, serving as monoatomic bridges via one carboxylate O‐atom (O9) bridging one apical Gd^III^ (Gd1) and one Ni^II^ ion of the {Ni_3_} ring (Ni1). The twelve aib^−^ ligands are found in their η^2^:η^1^:η^1^:μ_3_ binding mode, half of which direct the formation of the two {Ni_3_} triangles, with one carboxylate O‐atom further bridging to an apical Gd^III^ ion. The remaining aib^−^ ligands connect the [Ni_6_⊂Gd_5_] cage to the three Ni^II^ caps, but with one carboxylate O‐atom now bridging to a basal Gd^III^ ion. The connectivity to the three Ni^II^ caps is further supported by six η^1^:η^1^:μ‐OAc^−^ ions. All Gd^III^ ions are nine‐coordinate, the three basal ions adopting a spherical capped square antiprism geometry (Table S2), while the two apical ions conform to a spherical tricapped trigonal prism coordination sphere [28]. Similarly, the Ni^II^ ions are all six‐coordinate and adopt distorted octahedral geometries with {NO_5_} and {N_2_O_4_} coordination spheres for the two {Ni_3_} triangles and the three Ni^II^ caps, respectively. In detail, the basal angles surrounding the central atoms Ni1 and Ni2 deviate heavily from the theoretical values expected for an ideal O_h_ geometry, falling in the ∼77.8–97.5° and ∼80.0–110.2° range, respectively (Figure S1). Similarly, significant variations in the Ni─O bond lengths for Ni1 and Ni2 are observed, ranging between 2.03–2.17 Å and 2.04–2.07 Å, respectively, which further emphasise the distorted nature of their coordination geometries.

**FIGURE 3:**
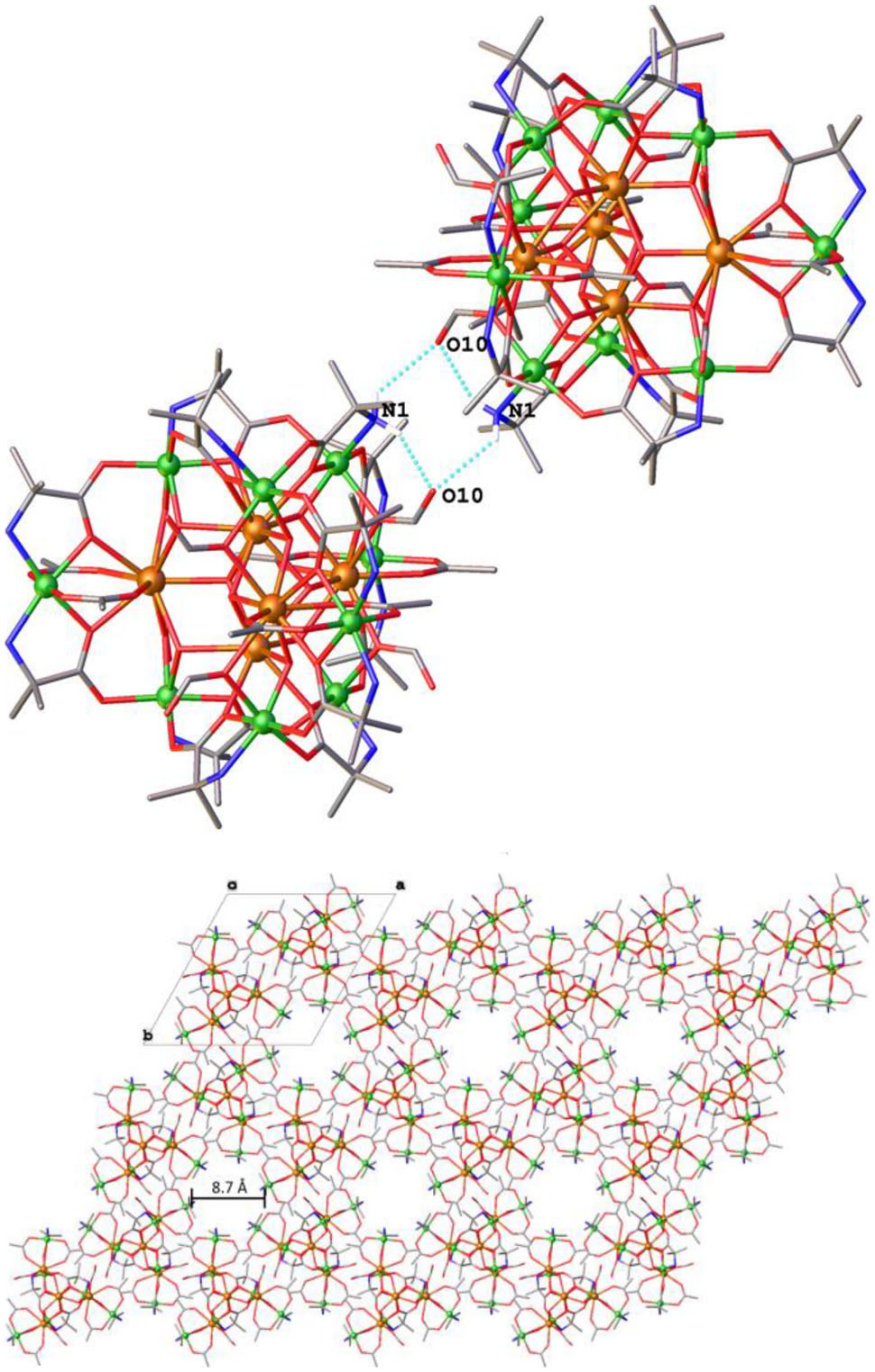
Intra‐ and intermolecular H‐bonds (light‐blue dashed lines) between neighboring HCOO^−^ and aib^−^ species in **1** (top). Packing of **1** in the *ab* plane giving rise to a honeycomb pattern featuring hexagonal channels with a diameter of ∼8.7 Å (bottom). Color scheme: Gd^III^, brown; Ni^II^, green; O, red; N, blue; C, grey. Hydrogen atoms omitted for clarity.

An extensive network of intermolecular hydrogen bonding between neighbouring HCOO^−^ and aib^−^ species dictates the packing of **1**. In each case, a HCOO^−^/aib^−^ pair connected via an intramolecular H‐bond (O10···N1, ∼2.88 Å, Figure S2) further H‐bonds to their symmetry equivalent counterparts on a neighbouring molecule (O10···N1, ∼3.08 Å), thus forming a parallelogram composed of two intramolecular and two intermolecular H‐bonds (Figure 3, top). Six of these parallelograms establish the link between neighbouring molecules, thus extending the connectivity between clusters along three directions within the *ab* plane. The result is an aesthetically pleasing honeycomb pattern featuring hexagonal channels with a diameter of ∼8.7 Å (Figure 3, bottom).

**FIGURE 4:**
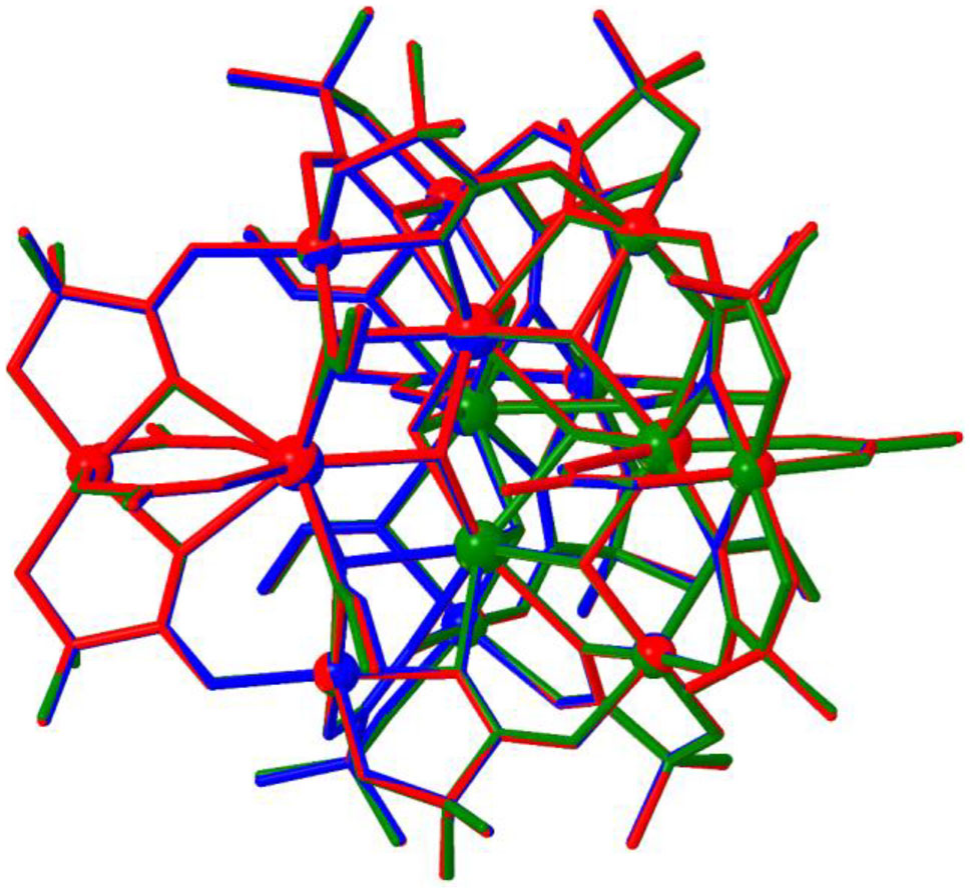
Overlay of the molecular structures of complexes **1** (red), **2** (green) and **3** (blue).

Complex **2** is isostructural to complex **1** where the five Gd^III^ centres have been replaced by five Y^III^ ions; thus, it will not be discussed further for the sake of brevity. For complex **3**, the metallic skeleton remains unchanged, the only difference being the presence of three bridging formates that have replaced three of the six η^1^:η^1^:μ acetates found in **1**, albeit the effect of this being deemed negligible regarding the structure of **3** (Figure 4). In the crystal lattice, the molecules of **3** are arranged in a honeycomb motif forming channels running down the *c* axis of the unit cell (Figure S3).

An extensive search of the Cambridge Structural Database yielded several compounds composed of a central {Gd_5_} trigonal bipyramid [29, 30, 31, 32]. Most notably, a [Cu_6_Gd_5_] [32] and two [Ni_12_Gd_5_] clusters [30, 31] have been reported, all of which contain the isostructural [M_6_⊂Gd_5_] metallic unit as in complexes **1**–**3**. As such, the main difference between these complexes lies within the number of metal caps present. Interestingly, the [Cu_6_Gd_5_] metallic unit features no further capping while the [Ni_12_Gd_5_] cluster is hexa‐capped. Thus, the tricapped {[Ni_6_⊂Gd_5_]Ni_3_} metallic unit present in complexes **1**–**3** extends the family of molecular clusters containing a central {Gd_5_} trigonal bipyramid, while displaying a new structural motif that falls in between the two extremes of uncapped and hexa‐capped [M_6_⊂Gd_5_] metallic units.

### 2.3 Magnetic Properties

**FIGURE 5:**
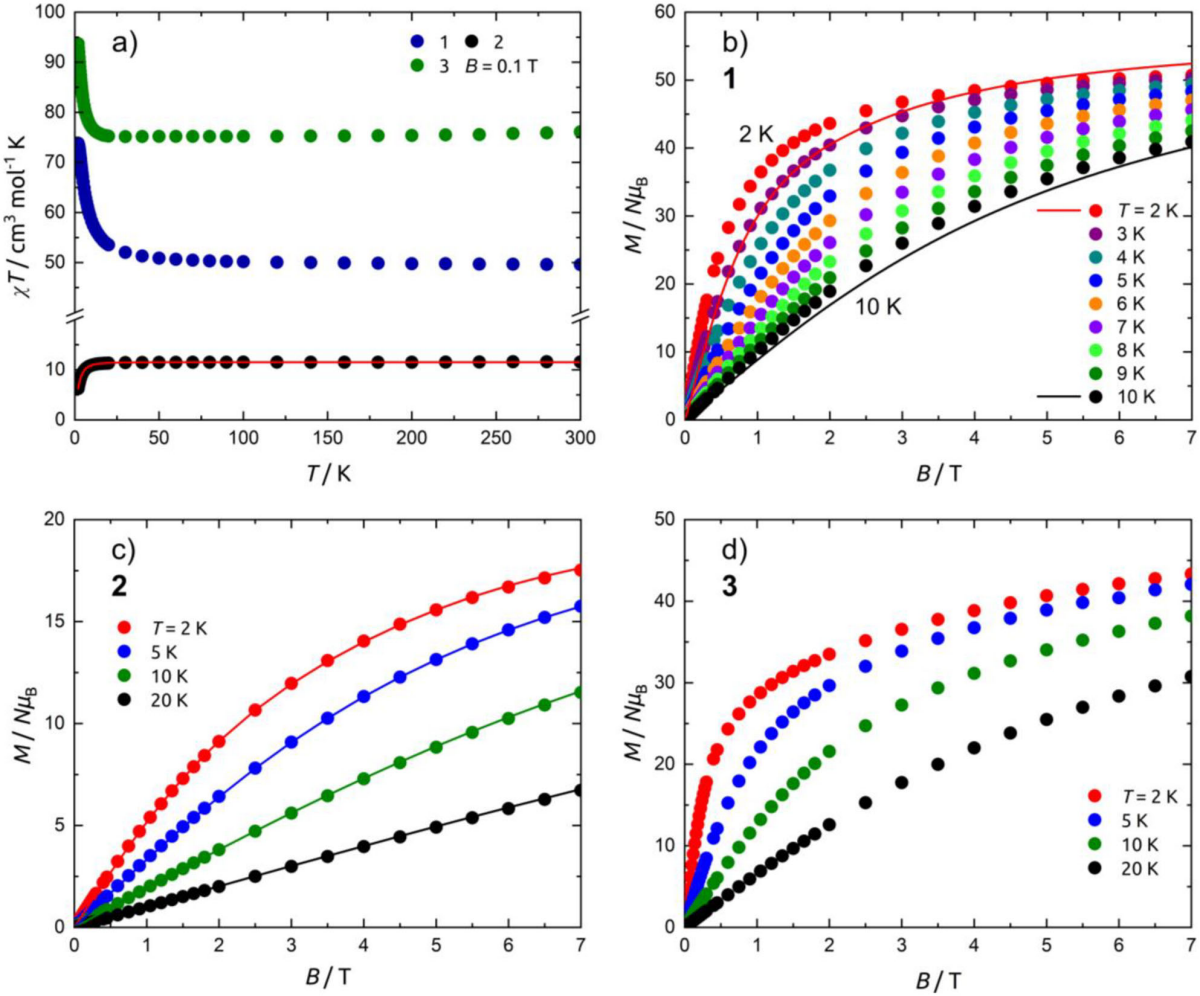
Temperature dependence of the *χ*_M_ *T* product for complexes **1**–**3** (a), collected under applied magnetic field of *B* = 0.1 T, and isothermal molar magnetization versus applied magnetic field for **1** (b) **2** (c) and **3** (d), respectively.

Direct current magnetic measurements were conducted on freshly prepared polycrystalline samples of **1**–**3** (Figure 5). The purity of all samples was established by means of PXRD comparison with the simulated data from the single‐crystal structure (Figure S4), as well as from energy‐dispersive x‐ray spectroscopy (Figure S5). Magnetic susceptibility data, *χ*_M_, were collected under an applied field of 0.1 T, in the 2–300 K temperature range. Field‐dependent isothermal magnetization, *M*, curves were recorded in the 0–7 Τ magnetic fields range for temperatures up to 20 K.

Room‐temperature *χ*_M_ *T* values of 49.6 cm^3^ mol^−1^ K for **1**, 11.5 cm^3^ mol^−1^ K for **2** and 76.1 cm^3^ mol^−1^ K for **3** were experimentally measured (Figure 5a). These values closely match the Curie constants expected for nine Ni^II^ and five Gd^III^ independent ions (48.4 cm^3^ mol^−1^ K), nine Ni^II^ independent ions (9.0 cm^3^ mol^−1^ K), and nine Ni^II^ and five Dy^III^ independent ions (76.3 cm^3^ mol^−1^ K), respectively.

For **2**, *χ*_M_ *T* remains almost constant down to ∼25 K, below which it decreases to 6.3 cm^3^ mol^−1^ K at 2 K (Figure 5a). This behavior can be well‐described by the single‐ion *s* = 1 Hamiltonian:

$$\;H=D_{\mathrm{Ni}}s_{z}^{2}+E_{\mathrm{Ni}}\left(s_{x}^{2}-s_{y}^{2}\right)-g_{\mathrm{Ni}}\mu_{B}Bs$$

The fitting of the experimental data performed using the software PHI [33], allows to additionally accommodate mean‐field interactions through a *zJ* term. The best fit of the magnetic data provided the values $g_{\mathrm{Ni}}$ = 2.26, $D_{\mathrm{Ni}}$ = 6.06 cm^−1^, $E_{\mathrm{Ni}}$ = 3.12 cm^−1^, and *zJ* = 0.02 cm^−1^, which indicates the presence of very weak antiferromagnetic correlations. The same model can satisfactorily describe the isothermal magnetization data (Figure 5c).

For **1**, *χ*_M_ *T* is nearly temperature independent down to ∼50 K, below which it increases rapidly to a maximum of 73.8 cm^3^ mol^−1^ K at 2 K. This rapid rise is attributed to ferromagnetic exchange interactions, which are absent in the Y analogue. Magnetization data further support the presence of ferromagnetism. At every temperature, the sum of the experimental magnetization of **2** and the calculated contribution of five non‐interacting Gd^ΙΙΙ^ ions lies below the experimental magnetization of **1** at low fields, an effect that becomes more pronounced as the temperature decreases (Figure 5b, representative curves shown for 2 and 10 K). This discrepancy provides strong evidence for dominant ferromagnetic correlations in **1**. For **3**, the room temperature value of *χ*_M_ *T* = 76.3 cm^3^ mol^−1^ K remains constant until ∼20 K, before gradually increasing to its maximum value of 93.7 cm^3^ mol^−1^ K at 2 K, with the later increase probably suggesting also the presence of dominant ferromagnetic interactions between the metal centers, similarly to **1**. No out‐of‐phase AC susceptibility signal was observed, indicating the absence of slow magnetic relaxation under the conditions investigated.

### 2.4 Magnetothermal Properties

**FIGURE 6:**
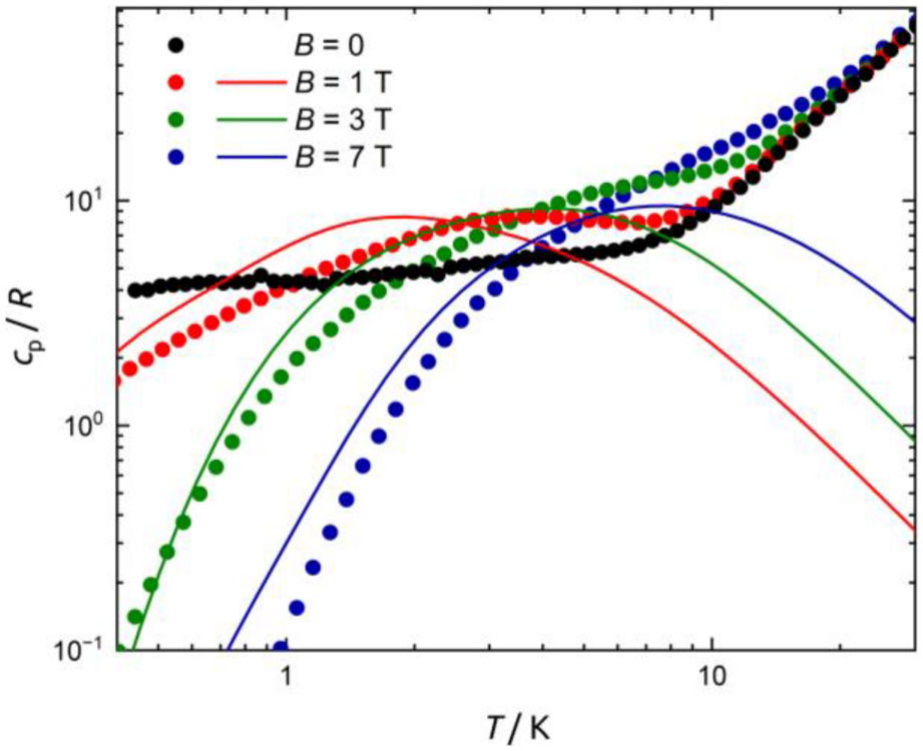
Heat capacity, *c*_p_, versus temperature at selected applied field values for **1**. Symbols are experimental data, while lines are theoretical simulations (see main text).

We evaluated the MCE of the gadolinium‐containing 3d–4f molecular cluster **1**, in which the highly isotropic Gd^ΙΙΙ^ ions are combined with Ni^II^ transition metal centrs. Heat capacity, *c*_p_, measurements were collected in the 0.3–30 K temperature range under selected applied magnetic fields up to 7 T (Figure 6). At temperatures above ∼10 K, *c*_p_ is dominated by the nonmagnetic lattice contribution, whereas at lower temperatures a broad Schottky‐type heat capacity emerges. Notably, this magnetic anomaly spans an unusually wide temperature range and becomes even broader as the applied magnetic field decreases. Such behavior likely reflects the combined effects of crystal‐field anisotropy and magnetic contributions of markedly different magnitudes.

To roughly disentangle the various magnetic contributions to the heat capacity of **1**, we simulated the response of nine independent Ni^II^ ions with $g_{\mathrm{Ni}}$ = 2.26, $D_{\mathrm{Ni}}$ = 6.06 cm^−1^, $E_{\mathrm{Ni}}$ = 3.12 cm^−1^, and *zJ* = 0.02 cm^−1^ parameter, as derived from the fit of the magnetic data of **2**, together with five non‐interacting Gd^ΙΙΙ^ ions. Although this simplified model neglects Ni─Gd and Gd─Gd exchange interactions, it reproduces the Schottky‐type anomalies reasonably well, particularly at the higher applied fields (3 and 7 T). At lower fields, the maximum of the experimental anomaly appears at higher temperature than the model prediction, indicating the presence of additional interactions. This shift is fully consistent with dominant ferromagnetic correlations, as already inferred from the magnetization data.

**FIGURE 7:**
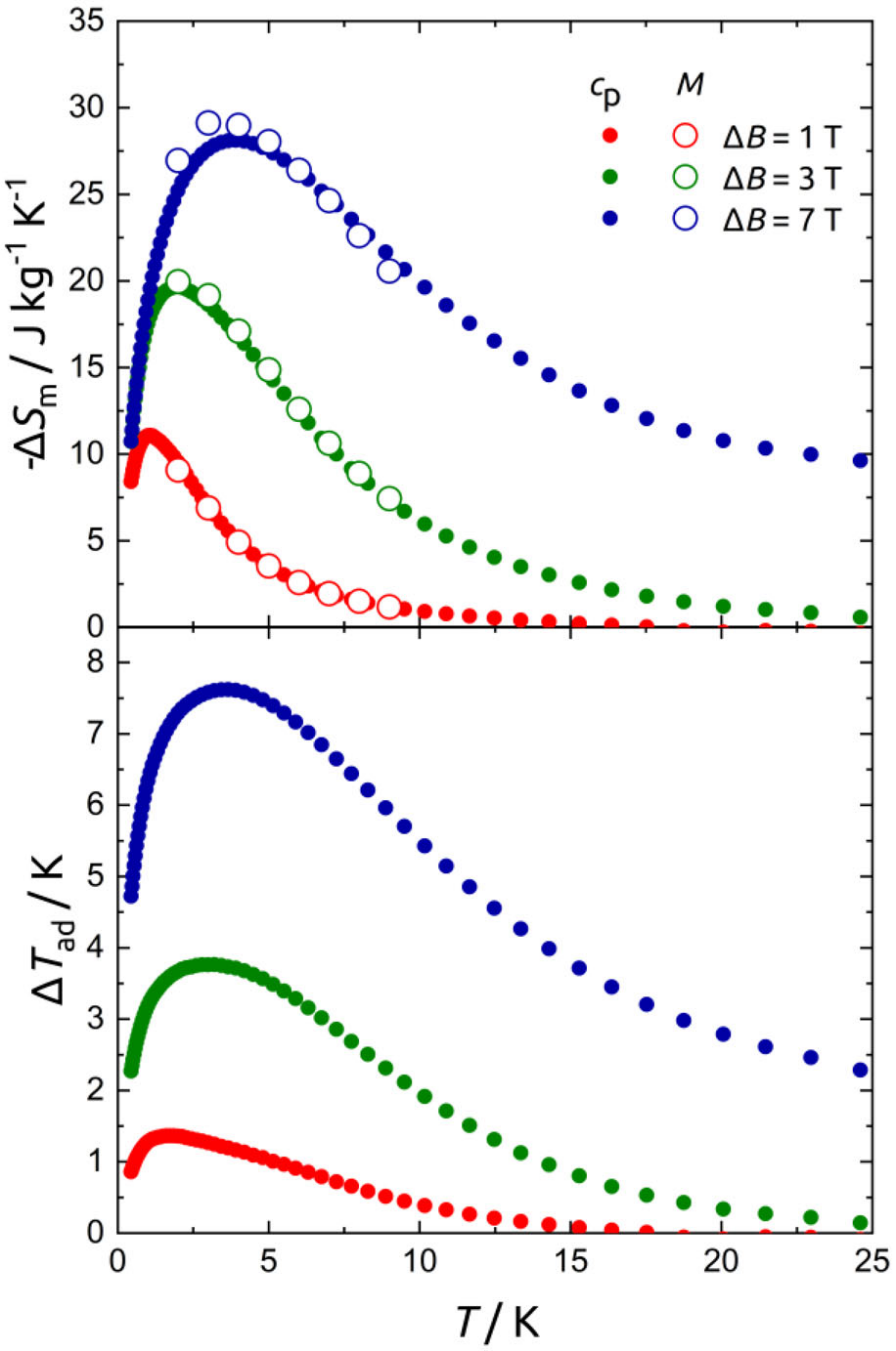
Magnetic entropy change, ∆*S*_m_, (top) and adiabatic temperature change, Δ*T*_ad_, (bottom) versus temperature for **1** at selected field changes, calculated from magnetization (empty symbols) and heat capacity (filled symbols) data.

The temperature dependence of the entropy, *S*, was obtained from the experimental *c*_p_ data, by applying the equation and is depicted in the inset of Figure S6. From the *S* data, we straightforwardly obtain the MCE figures of merit, namely the magnetic entropy change, ∆*S*_m_, and the adiabatic temperature change, ∆*T*_ad_, both obtained as the differences between the entropy curves for any field change ∆*B *= *B*–0 (Figure 7). As can be seen, −∆*S*_m_ reaches a maximum of 28.1 J kg^−1^ K^−1^ at *T* = 3.9 K for ∆*B =* 7 T, whereas the maximum of ∆*T*_ad_ amounts to 7.6 K at 3.6 K for the same field change.


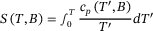




The magnetic entropy change, ∆*S*_m_, was also determined from the magnetization data (Figure 5b) using the Maxwell relation yielding identical results across the temperature range where both methods overlap (Figure 7). The convergence of the two approaches underscores the validity of the MCE determined for **1**.

$$\Delta S_{m}\left(T,\Delta B\right)=\int_{0}^{B}{\left(\frac{\partial M}{\partial T}\right)}_{B}dB,$$

The observed maximum value of −∆*S*_m_ = 28.1 J kg^−1^ K^−1^ corresponds to ∼55% of the total available magnetic entropy (51.5 J kg^−1^ K^−1^). This total entropy is derived from the expected contribution of the 9 Ni^II^ (*s* = 1) and 5 Gd^ΙΙΙ^ (*s* = 7/2) spins, which together provide an entropy content of 9 × ln(3) + 5 × ln(8) per molecular unit. Although reaching ∼55% of the available entropy under ∆*B =* 7 T may appear modest when compared with some of the highest‐performing molecular magnetic refrigerants, compound **1** distinguishes itself by sustaining large −∆*S*_m_(*T*, ∆*B*) values over an exceptionally broad temperature range, a behavior likewise reflected in its ∆*T*_ad_(*T*, ∆*B*) response. Notably, −∆*S*_m_ = 9.6 J kg^−1^ K^−1^ at *T* = 25 K, which still exceeds one third of the maximum value, whereas the corresponding adiabatic temperature change is also sizeable, ∆*T*_ad_ = 2.3 K at the same high temperature (Figure 7).

The broad −∆*S*_m_ profile also implies a large relative cooling power (RCP = |∆*S*_m_|×*δT*_FWHM_, where *δT*_FWHM_ is the full width at half maximum), which reaches 394 J kg^−1^ for ∆*B =* 7 T and compares well with most reported Ni/Gd clusters (Table 1). This performance originates from the broad Schottky‐like anomaly (Figure 6), which results from the combined effects of crystal‐field anisotropy and magnetic interactions of different energy scales within the molecule, influencing both the Ni^II^ and Gd^ΙΙΙ^ centres.

**TABLE 1:** Comparison of Ni/Gd‐based magnetocaloric refrigerants. Reported values include magnetic entropy change (−∆*S*_m_) for ∆*B* = 7 T, temperature at which −∆*S*_m_ is maximum, and relative cooling power (RCP) for ∆*B* = 7 T.

Compound	−∆*S*m (J kg^−1^ K^−1^)	*T* (K)	RCP (J kg^−1^)	Ref.
[Gd_42_Ni_10_(μ_3_‐OH)_68_(CO_3_)_12_(CH_3_COO)_30_(H_2_O)_70_]·(ClO_4_)_24_·80H_2_O	38.2	2.0	—	[16]
[Gd_152_Ni_14_Cl_24_(CO_3_)_42_(OAc)_48_(H_2_O)_138_(OH)_308_]·Cl_20_·(H_2_HEIDA)_10_·20H_2_O	52.7	3.5	569	[18]
[Ni_6_Gd_6_(OH)_2_(O_3_PCH_2_Ph)_6_(O_2_C^t^Bu)_16_(MeCO_2_H)_2_]·4MeCN	26.5	3.0	—	[34]
[Gd_36_Ni_12_(CH_3_COO)_18_(μ_3_‐OH)_84_(μ_4_‐O)_6_(H_2_O)_54_(NO_3_)Cl_2_](NO_3_)_6_Cl_9_·30H_2_O	36.3	3.0	—	[35]
[Ni^II^_8_Gd_4_(OH)_8_(OAc)_4_(L’)_8_(H_2_O)_4_]^.^24H_2_O	18.1	3.7	298	[36]
[Ni_2_Gd(L)_6_](NO_3_)	13.7	4.0	178	[37]
[NiGd(HL)_2_(NO_3_)_3_]	5.7	7.0	68	[38]
[Ni(H_2_O)_6_][Gd(oda)_3_]·3H_2_O	28.5	2.0	242	[39]
[Ni_64_Gd_96_(μ_3_‐OH)_156_(IDA)_66_(DMPA)_12_(CH_3_COO)_48_(NO_3_)_24_(H_2_O)_64_]Cl_24_·35CH_3_OH·120H_2_O	42.8	3.0	—	[40]
[Na_2_(Gd_78_Ni_64_(IDA)_58_(OAc)_2_(SiO_4_)_6_(Cl)_4_(μ_2_‐OH)_4_(μ_4_‐O)_4_(μ_3_‐O)_38_(μ_3_‐OH)_126_(H_2_O)_82_]·(Cl)_4_(H_2_O)_37_	40.6	3.0	428	[41]
[Ni_36_Gd_102_(OH)_132_(mmt)_18_(dmpa)_18_(H_2_dmpa)_24_(CH_3_COO)_84_(SO_4_)_18_(NO_3_)_18_(H_2_O)_30_]·Br_6_(NO_3_)_6_·(H_2_O)_130_·(CH_3_OH)_60_	41.3	2.0	—	[42]
[Ni_5_Gd_8_(μ_3_‐OH)_7_(μ‐OH_2_)(O_3_P‐^t^Bu)_6_(O_2_C‐^t^Bu)_15_(MeCN)]	30.6	3.0	386	[43]
[Gd_22_Ni_22_(DTA)_21_(SO_4_)_4_(OH)_48_(OCH_3_)_4_](NO_3_)_6_·(H_2_O)_50_	46.0	2.0	419	[44]
[Ni_21_Gd_20_(OH)_24_(IDA)_21_(DPGA)_6_(C_2_O_4_)_3_(NO_3_)_6_(CH_3_COO)_3_(H_2_O)_12_]·Br_5_·(NO_3_)_4_·20CH_3_OH·30H_2_O	34.8	3.0	—	[45]
[Ni_9_Gd_5_(OH)_9_(aib)_12_(OAc)_6_(HCOO)_6_]	28.1	3.9	394	This work

## 3 Conclusion

In summary, we have synthesized and structurally characterised three new isostructural tetradecanuclear 3d–4f clusters, {Ni_9_Ln_5_} (Ln = Gd, Y, Dy), constructed around a trigonal bipyramidal {Ln_5_} core encapsulated within a triply edge‐capped {[Ni_6_]Ni_3_} trigonal prism. This architecture represents a well‐defined intermediate stage in the structural evolution of the {M_6_⊂Gd_5_} metallic unit, bridging previously reported uncapped and hexa‐capped analogues, and highlighting its modular growth potential.

Magnetic measurements show that the Gd and Dy analogues exhibit dominant ferromagnetic interactions, in contrast to the weak antiferromagnetic interactions observed for the Y derivative. Heat‐capacity and magnetization studies further reveal a sizable magnetocaloric effect for the Gd compound, reaching −∆*S*_m_ = 28.1 J kg^−1^ K^−1^ and ∆*T*_ad_ = 7.6 K for ∆*B* = 7 T, while maintaining a comparatively large response across an unusually broad temperature range. Such a broadened operational window is advantageous for practical adiabatic demagnetization cycles, where the accessible temperature span is often as critical as the absolute maxima of −∆*S*_m_ and ∆*T*_ad_.

## Conflicts of Interest

The authors declare no conflicts of interest.

## Supporting information



Further synthetic and crystallographic details, structural visualizations, pXRD diagrams, EDS spectra, TGA analysis, IR spectra, entropy curves and SHAPE calculations for **1**, **2** and **3** are included in the Supporting Information. The authors have cited additional references within the Supporting Information [46, 47, 48].
**Supporting File**: chem71250‐sup‐0001‐SuppMat.docx

**Supporting File**: chem71250‐sup‐0002‐cif.zip

## Data Availability

Deposition Number(s) 2533099 (**1**), 2533100 (**2**), and 2533101 (**3**) contain the supplementary crystallographic data for this paper. These data are provided free of charge by the joint Cambridge Crystallographic Data Centre and Fachinformationszentrum Karlsruhe. Magnetic and magnetothermal data for compounds **1**, **2** and **3** can be retrieved from DIGITAL.CSIC at http://doi.org/10.20350/DIGITALCSIC/18241.
