# Supplementary material for: Tetradecanuclear {Ni9Ln5} Clusters Featuring Encapsulated {Ln5} Trigonal Bipyramids: Modular 3d‐4f Architectures With Broad‐Range Magnetocaloric Response
Source: Chemistry. 2026 Jun 8;32(35):e71250. doi: 10.1002/chem.71250 (PMC13587641; doi:10.1002/chem.71250)
Supplement: Supplementary file 1 [file CHEM-32-e71250-s001.docx]

Chemistry – A European Journal

Supporting Information

**Tetradecanuclear {Ni_9_Ln_5_} clusters featuring encapsulated {Ln_5_} trigonal bipyramids: Modular 3*d*-4*f* architectures with broad-range magnetocaloric response**

Thomais G. Tziotzi, Evangelia G. Asmargiannaki, Ben Thamm, Scott J. Dalgarno, Emmanouil K. Charkiolakis, Marc Ubach I Cervera, David Gracia, Marco Evangelisti,* and Constantinos J. Milios*

**General**

All chemicals were obtained from commercial suppliers and were used without further purification/treatment. Powder XRD measurements were collected on freshly prepared samples of **1**-**3** on a PANanalytical X’Pert Pro MPD diffractometer. Magnetisation data were collected on a QD-MPMS3 SQUID magnetometer equipped with a 7 T magnet. Diamagnetic corrections were applied using Pascal's constants. Heat capacity measurements were carried out on a QD-PPMS equipped with a 9 T magnet and a ^3^He cryostat, using a thin pressed pellet (ca. 1 mg) of polycrystalline samples of **1** and **3**, thermalized by ca. 0.2 mg of Apiezon N grease, whose contribution was subtracted using a phenomenological expression. EDS measurements were performed on a JEOL Scanning Electron Microscope. FTIR−ATR spectra were recorded on a JASCO FTIR-6600 spectrometer at the University of Zaragoza. Thermogravimetric analyses (TGA) were performed on a TA Instruments TGA 5500 thermogravimetric analyzer under N₂ atmosphere at a heating rate of 5 °C min⁻¹ at the University of Crete. Single crystal X-ray diffraction data were collected on a Bruker D8 VENTURE diffractometer (University of Crete), equipped with a PHOTION II CPAD detector. Using Olex2,^[28]^ single crystal X-ray structures were solved with the SHELXT^[29]^ structure solution program using Intrinsic Phasing and refined with the SHELXL^[30]^ refinement package using least squares minimisation. Data collection parameters and structure solution and refinement details are listed in Table S1. Full details can be found in the CIF files with CCDC reference numbers 2533099, 2533100, and 2533101, for complex **1, 2** and **3**, respectively.

**Syntheses of the complexes**

Synthesis of **1**∙12MeCN

Ni(HCOO)_2_·2H_2_O (93 mg, 0.5 mmol), Gd(OAc)_3_·4H_2_O (203 mg, 0.5 mmol), aibH (52 mg, 0.5 mmol) and NEt_3_ (0.5 mmol) were added in a mixture of solvents MeCN/MeOH (15 ml, 1:1) and then transferred to a Teflon-lined autoclave. The autoclave was kept in an oven at 120°C for 30 h, and then allowed to return gradually at room temperature, forming light-blue crystals of **1**^.^12MeCN in ～35 % yield. The crystals were collected by filtration and dried under vacuum. Elemental analysis (%) calcd for **1**^.^5MeCN: C 25.21, H 3.91, N 6.85; found: C 25.09, H 3.77, N 6.76.

Synthesis of **2**∙12MeCN and **3**∙12MeCN

Complex **2**∙12MeCN and **3**∙12MeCN were prepared in a similar manner to **1**^.^12MeCN using Y(OAc)_3_^.^4H_2_O and Dy(OAc)_3_^.^4H_2_O, respectively for **2**^.^12MeCN and **3**^.^12MeCN, instead of Gd(OAc)_3_^.^4H_2_O. Elemental analysis (%) calcd for **2**^.^4MeCN: C 27.56, H 4.30, N 7.24; found: C 27.45, H 4.19, N 7.16. Elemental analysis (%) calcd for **3**^.^4MeCN: C 24.61, H 3.93, N 6.47; found: C 23.52, H 3.79, N 6.35.

**Table S1**. Crystal data and structure refinement for complexes **1**∙12MeCN, **2**∙12MeCN and **3**∙12MeCN.

|  | **1**∙12MeCN | **2**∙12MeCN | **3**∙12MeCN |
| --- | --- | --- | --- |
| Empirical formula | C_87_H_156_Gd_5_N_24_Ni_9_O_57_ | C_87_H_156_N_24_Ni_9_O_57_Y_5_ | C_87_H_159_Dy_5_N_24_Ni_9_O_57_ |
| Formula weight | 3764.99 | 3423.29 | 3794.26 |
| Temperature/K | 220(2) | 220(2) | 200(2) |
| Crystal system | Hexagonal | Hexagonal | Hexagonal |
| Space group | *P6_3_/m* | *P6_3_/m* | *P6_3_/m* |
| *a*/Å | 19.4891(7) | 19.6135(4) | 19.3163(6) |
| *b*/Å | 19.4891(7) | 19.6135(4) | 19.3163(6) |
| *c*/Å | 20.4162(11) | 20.2667(8) | 20.3032(9) |
| *α*/° | 90 | 90 | 90 |
| *β*/° | 90 | 90 | 90 |
| *γ*/° | 120 | 120 | 120 |
| Volume/Å^3^ | 6715.7(6) | 6751.9(4) | 6560.6(5) |
| *Z* | 2 | 2 | 2 |
| *ρ*_calc_ g/cm^3^ | 1.862 | 1.684 | 1.921 |
| *μ*/mm^‑1^ | 3.754 | 3.438 | 4.163 |
| *F*(000) | 3748.0 | 3498.0 | 3774.0 |
| Crystal size/mm^3^ | 0.34 × 0.3 × 0.26 | 0.32 × 0.3 × 0.24 | 0.4 × 0.36 × 0.32 |
| Radiation | MoKα (λ = 0.71073) | MoKα (λ = 0.71073) | MoKα (λ = 0.71073) |
| 2Θ range for data collection/° | 3.99 to 58.344 | 4.02 to 58.39 | 4.67 to 58.806 |
| Index ranges | -25 ≤ h ≤ 26, -26 ≤ k ≤ 26,  -27 ≤ l ≤ 27 | -26 ≤ h ≤ 26, -26 ≤ k ≤ 25,  -27 ≤ l ≤ 26 | -26 ≤ h ≤ 26, -25 ≤ k ≤ 26, -27 ≤ l ≤ 27 |
| Reflections collected | 67871 | 72840 | 42642 |
| Independent reflections | 6212 | 6252 | 6158 |
| Data/restraints/parameters | 6212/19/254 | 6252/20/278 | 6158/21/262 |
| Goodness-of-fit on F^2^ | 1.027 | 1.044 | 1.037 |
| Final *R* indexes [*I*>=2σ (I)] | R_1_ = 0.0315, wR_2_ = 0.0660 | R_1_ = 0.0478, wR_2_ = 0.1283 | R_1_ = 0.0219, wR_2_ = 0.0513 |
| Largest diff. peak/hole / e Å^-3^ | 0.90/-0.90 | 1.15/-0.75 | 1.34/-1.33 |

**Figure S1.** The severely distorted octahedral geometries of the Ni1 (left) and Ni2 atom (right). Colour scheme: Ni^II^, green; O, red; N, blue.

**Figure S2.** Intramolecular H-bonds (light-blue dashed lines) between neighbouring HCOO^−^ ions and aib^−^ ligands in complex **1**. Selected hydrogen atoms are shown on amine groups for clarity. Colour scheme: Gd^III^, brown; Ni^II^, green; O, red; N, blue; C, grey; H, white.

**Figure S3.** The voids (red) present within the packing of **3**. Colour scheme: Dy^III^, turquoise; Ni^II^, green; O, red; N, blue; C, grey. Hydrogen atoms omitted for clarity.

**Figure S4.** Powder XRD pattern comparison for **1** - **3** (top-to-bottom) and their simulated PXRD patterns.

**Figure S5.** EDS spectra for **1** – **3** (top to bottom).

**Figure S6.** Entropy *S* *versus* temperature for selected applied field values for **1**.

**Figure S7.** Thermal analysis of freshly prepared samples of **1** – **3**. For complex **1** there is a weight loss of 8.4% in the 25 – 135 ^o^C temperature range, corresponding to ~ 8 MeCN molecules, while for complex **2** a weight loss of 11.1% in the same temperature region occurs (corresponding to ~ 9 MeCN molecules). Complex **3** displays a weight loss of 5.6 % in the 25-135 ^o^C range, with a concomitant weight loss of 4.4% in the 135-157 ^o^C range (total weight loss of 10.0%, corresponding to ~ 9 MeCN molecules).

**Figure S8.** FTIR−ATR spectra for complexes **1**-**3**.

**Table S2.** SHAPE calculations for Gd^III^, Y^III^ and Dy^III^ centres in **1**, **2** and **3**.

Complex **1**: Gd1, Gd2

| **Structure [ML_9_]** | **Symmetry** | **Geometry** | **Gd1** | **Gd2** |
| --- | --- | --- | --- | --- |
| EP-9 | D_9h_ | Enneagon | 35.713 | 37.861 |
| OPY-9 | C_8v_ | Octagonal pyramid | 21.193 | 21.425 |
| HBPY-9 | D_7h_ | Heptagonal bipyramid | 19.927 | 21.466 |
| JTC-9 | C_3v_ | Johnson triangular cupola J3 | 14.719 | 16.376 |
| JCCU-9 | C_4v_ | Capped cube J8 | 11.683 | 11.712 |
| CCU-9 | C_4v_ | Spherical-relaxed capped cube | 10.376 | 10.110 |
| JCSAPR-9 | C_4v_ | Capped square antiprism J10 | 1.899 | 2.580 |
| **CSAPR-9** | **C_4v_** | **Spherical capped square antiprism** | **0.769** | 1.255 |
| JTCTPR-9 | D_3h_ | Tricapped trigonal prism J51 | 3.386 | 2.944 |
| **TCTPR-9** | **D_3h_** | **Spherical tricapped trigonal prism** | 1.496 | **0.198** |

Complex **2**: Y1, Y2

| **Structure [ML_9_]** | **Symmetry** | **Geometry** | **Y1** | **Y2** |
| --- | --- | --- | --- | --- |
| EP-9 | D_9h_ | Enneagon | 37.802 | 35.072 |
| OPY-9 | C_8v_ | Octagonal pyramid | 21.437 | 20.654 |
| HBPY-9 | D_7h_ | Heptagonal bipyramid | 21.454 | 20.119 |
| JTC-9 | C_3v_ | Johnson triangular cupola J3 | 16.248 | 14.589 |
| JCCU-9 | C_4v_ | Capped cube J8 | 11.671 | 11.306 |
| CCU-9 | C_4v_ | Spherical-relaxed capped cube | 10.062 | 10.180 |
| JCSAPR-9 | C_4v_ | Capped square antiprism J10 | 2.616 | 1.701 |
| **CSAPR-9** | **C_4v_** | **Spherical capped square antiprism** | 1.289 | **0.723** |
| JTCTPR-9 | D_3h_ | Tricapped trigonal prism J51 | 2.988 | 3.311 |
| **TCTPR-9** | **D_3h_** | **Spherical tricapped trigonal prism** | **0.229** | 1.553 |

Complex **3**: Dy1, Dy2

| **Structure [ML_9_]** | **Symmetry** | **Geometry** | **Dy1** | **Dy2** |
| --- | --- | --- | --- | --- |
| EP-9 | D_9h_ | Enneagon | 37.789 | 36.152 |
| OPY-9 | C_8v_ | Octagonal pyramid | 21.439 | 21.672 |
| HBPY-9 | D_7h_ | Heptagonal bipyramid | 21.471 | 19.929 |
| JTC-9 | C_3v_ | Johnson triangular cupola J3 | 16.351 | 14.804 |
| JCCU-9 | C_4v_ | Capped cube J8 | 11.651 | 11.602 |
| CCU-9 | C_4v_ | Spherical-relaxed capped cube | 10.036 | 10.293 |
| JCSAPR-9 | C_4v_ | Capped square antiprism J10 | 2.610 | 1.861 |
| **CSAPR-9** | **C_4v_** | **Spherical capped square antiprism** | 1.277 | **0.724** |
| JTCTPR-9 | D_3h_ | Tricapped trigonal prism J51 | 2.983 | 3.303 |
| **TCTPR-9** | **D_3h_** | **Spherical tricapped trigonal prism** | **0.221** | 1.586 |
